# Spontaneous regression of extradural intraspinal cysts in a dog: a case report

**DOI:** 10.1186/s12917-019-2152-x

**Published:** 2019-11-06

**Authors:** Marília de Albuquerque Bonelli, Ronaldo Casimiro da Costa

**Affiliations:** Department of Veterinary Clinical Sciences, College of Veterinary Medicine, The Ohio State University, 601 Vernon Tharp St., Columbus, OH 43210 USA

**Keywords:** Juxtafacet cysts, Synovial cyst, Lumbar, Magnetic resonance imaging

## Abstract

**Background:**

Extradural intraspinal cysts are fluid accumulations that appear to be associated with increased motion at vertebral joints.

**Case presentation:**

We report the spontaneous regression of lumbar and lumbosacral cysts (presumably synovial cysts) and the unusual occurrence of an S1–2 extradural intraspinal cyst in a dog. The dog presented with lumbosacral pain. Six extradural intraspinal cysts were observed on high-field magnetic resonance imaging from L5–6 to S1-S2. The cysts between L5–6 and L7-S1 ranged from 0.12 to 0.44cm^2^ at their largest area. The largest cyst was located at S1–2 (left), measuring 0.84 cm^2^ at its largest view. The dog was medically managed. A follow-up magnetic resonance imaging scan was obtained 3.5 years after the first imaging. All cysts except the one at S1–2 had reduced in size. Mean reduction in size was 59.6% (35–81%).

**Conclusions:**

In summary, we report a case with multiple extradural intraspinal cysts that underwent spontaneous regression of all but one cyst during a 3.5-year follow-up period. Whether this is a single occurrence, or is part of the natural history of these cysts in the lumbosacral region of dogs, remains to be established. Spontaneous regression of intraspinal cysts had not been described in dogs.

## Background

Extradural intraspinal cysts, sometimes called juxtafacet cysts, are collections of fluid (or fluid-like material) usually in close proximity to vertebral joints. There are two main types of extradural cysts that have been described in dogs: synovial cysts and ganglion cysts [1]. This distinction is determined via histopathology, where synovial cysts are characterized by a synovial lining and fluid content and ganglion cysts have no synovial lining and myxoid content [2–4]. There is no clinical distinction between them and both may be referred to as extradural intraspinal cyst or juxtafacet cysts [1]. The pathophysiology of how these cysts develop has not been well established, but it is thought that their development is related to increased motion and/or instability between two adjacent vertebrae [1, 2].

Extradural intraspinal cysts have been reported throughout the vertebral column in dogs, but there have been no reported cases of cysts at S1-S2 [2, 5–7]. Clinical signs vary according to location of the cyst and structures being compressed, e.g.: nerve roots or spinal cord, but there is a predominance of localized pain upon palpation of the vertebral column [4–6, 8]. Presumptive diagnosis is best achieved via MR imaging studies, where the cysts are well circumscribed lesions hyperintense on T2W images and hypo or isointense on T1W images [1]. The preferred method of treatment is surgery to remove the cysts when these are considered symptomatic [2, 6]. There have been reports of spontaneous regression of extradural intraspinal cysts in humans, but the authors are unaware of previously reported cases of extradural intraspinal cyst regression in dogs [9].

Although it is generally accepted that surgery is the treatment of choice, the natural history and progression of extradural cysts has yet to be described. The aim of the present report is to describe the case of a German shepherd dog with extradural intraspinal cysts in the lumbar region of the vertebral column that underwent spontaneous regression.

## Case presentation

A 1.8-year-old male castrated German shepherd dog presented with a history of pain in the lumbar/lumbosacral region. The dog had been previously diagnosed with cervical spondylomyelopathy (CSM) confirmed with magnetic resonance imaging (MRI) and was being treated intermittently with prednisone for the past 3 months. At the time of CSM diagnosis, findings on cervical MRI included moderate compression of the spinal cord caused by bony proliferation of the articular processes and ligamentum flavum hypertrophy at C5–6.

On neurological examination, tetraparesis and proprioceptive ataxia were observed. The weakness in the pelvic limbs seemed more pronounced. Delayed conscious proprioceptive positioning in both pelvic limbs (worse in the right) and a decreased flexor reflex on the left thoracic limb were noted. The patellar reflexes were considered to be increased (with clonus in the left pelvic limb), and there was also increased extensor tone in all four limbs. Pain was observed on palpation at L6-L7 and L7-S1 and upon dorsal flexion of the tail. Based on neurologic examination, the dog was deemed to have a cervical myelopathy (compatible with the previous diagnosis of cervical spondylomyelopathy) and a lumbosacral radiculomyelopathy.

Magnetic resonance imaging of the lumbosacral region (L2 to S1–2) of the vertebral canal was performed to evaluate the pain noticed on palpation of the lumbar/lumbosacral spine. The MRI was performed using a 3 T scanner with the dog placed in dorsal recumbency. T2-weighted (W) images were obtained in sagittal, transverse, and dorsal planes. Pre- and post-contrast T1W images were obtained in sagittal and transverse planes.

Six cystic structures were observed within the vertebral canal at L5–6 on the left (0.20cm^2^), at L6–7 bilaterally (0.12cm^2^ on left and 0.25cm^2^ on right), at L7-S1 bilaterally (0.44cm^2^ on left and 0.22cm^2^ on right), and S1–2 on the left side (0.84cm^2^). Sizes are given as area on the largest view on sagittal or transverse T2W images. These cysts were hyperintense on T2W images and isointense on T1W images to the surrounding soft tissue. Rim enhancement was observed on T1W post-contrast images after gadolinium administration. The L7-S1 cysts were compressing the conus medullaris bilaterally. The cyst at S1–2 appeared to be causing mild compression of nerve roots. Other findings included arthrosis of the articular processes at L6–7 and L7-S1 and lumbarisation of S1.

Three years and six months after the initial lumbar MRI, the patient returned for re-evaluation of the previously observed changes. In the time elapsed from the first scan, the dog had been intermittently treated with prednisone due to the concurrent CSM, but had been off medications for the previous 10 months.

Main findings from the neurological exam at that time included generalized muscle atrophy (worse in thoracic limbs). There was moderate to marked tetraparesis with proprioceptive ataxia in all four limbs (left side worse), both noticeably worse in the pelvic limbs. Conscious proprioceptive positioning was decreased in the thoracic limbs and markedly decreased in the pelvic limbs, with the left worse than the right. The patellar reflex was considered increased and with clonus, and the flexor reflex was considered decreased in all but the right pelvic limb. Notably, at this time, there was no pain noticed on spinal palpation of the lumbar/lumbosacral region.

An MRI was performed of the lumbosacral spine (L5 to S1–2) also using a 3 T scanner and sagittal and transverse T1W and T2W images were obtained. A follow-up MRI of the cervical vertebral column was also obtained for evaluation of the concurrent CSM.

**Fig. 1:**
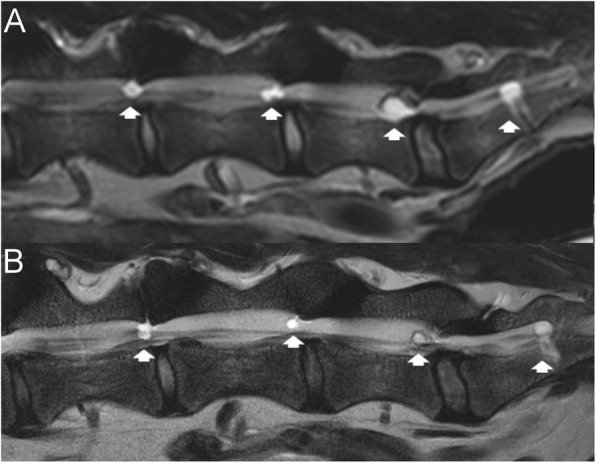
Initial (**a**) and 3.5-year follow-up (**b**) left parasagittal T2-weighted magnetic resonance imaging (MRI) of extradural intraspinal cysts in a German Shepherd dog. Cysts can be seen at L5–6, L6–7, L7-S1, and S1–2. All cysts except the one at S1–2 are smaller on the follow-up MRI

**Fig. 2:**
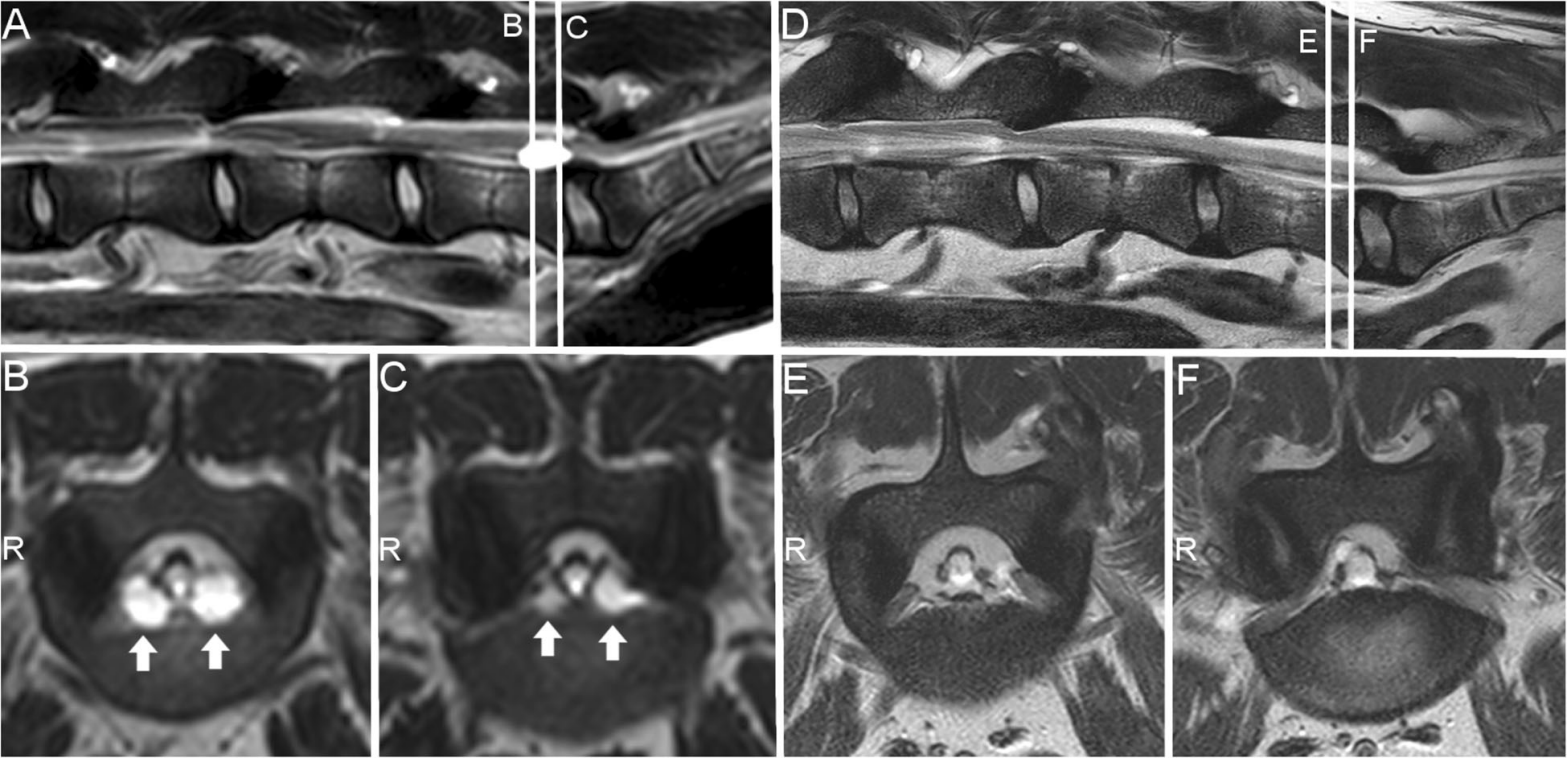
Initial (**a**, **b**, **c**) and 3.5-year follow-up (**d**, **e**, **f**) T2-weighted magnetic resonance imaging (MRI) of extradural intraspinal cysts in a German Shepherd dog. **a**: left parasagittal image showing a large extradural cyst at L7-S1 seen at the first lumbosacral MRI. The level at which the transverse images for **b** and **c** were obtained is represented by lines identified with corresponding letters. **b**: transverse image showing bilateral cysts (arrows) at L7. **c**: transverse image obtained at the level of the endplate of L7 showing the same cysts seen on **b**. **d**: left parasagittal image on follow-up showing at which level the transverse images for **e** and **f** were obtained (corresponding lines). Note the large cyst seen on **a** is no longer visible in this location. **e**, **f**: transverse images at approximately the same level as **b** and **c**. Note there was a reduction in size of both cysts. R, right

Findings of interest included reduction in size of all the cysts except at S1–2, which remained virtually the same size (Fig. 1). Most notable was the reduction of the left cyst at L7-S1 (81% reduction) (Fig. 2). Area on the largest view on sagittal or transverse T2W images of the cysts were: 0.13cm^2^ (left) at L5–6, 0.05cm^2^ (left) and 0.12cm^2^ (right) at L6–7, 0.08cm^2^ (left) and 0.06cm^2^ (right) at L7-S1, and 0.83cm^2^ at S1–2 (left). Overall, there was a mean reduction of 59.6% (range: 35–81%) in cyst area between the initial and follow-up lumbar MRI.

On cervical MRI, there was subjective worsening of the main compressive lesion at C5–6 due to osseous proliferation of the articular processes and ligamentum flavum hypertrophy.

## Discussion and conclusions

This case report describes the spontaneous regression of extradural intraspinal cysts in the lumbosacral region of the vertebral column of a large-breed dog observed on follow-up MRI 3.5 years after the first MRI study, as well as the unusual occurrence of a cyst at S1-S2. To the authors’ knowledge, this is the first report of spontaneous regression of extradural intraspinal cysts in a dog. It is also the first reported case of an extradural intraspinal cyst at S1-S2 in a dog.

In humans, it is rare for extradural intraspinal cysts to regress only with conservative management, but it has been reported [9–11]. There are mainly three hypotheses for regression: restoration of stability, spontaneous rupture, and anti-inflammatory use [12]. Stability would result from a natural progression of joint and disc degeneration, disc space collapse, and subsequent loss of motion, which would then allow the cysts to regress. Spontaneous rupture of the cysts has been associated with reports of inflammatory reactions and worsening of clinical signs, but the possibility of microruptures has been considered due to findings of adhesions between the cysts and dura mater [9]. The third hypothesis is based on regression observed after the use of nonsteroidal anti-inflammatory drugs [12].

In the present case, progressive degenerative joint disease may have played a role in the regression of the cysts. Whether or not leakage occurred is impossible to evaluate, but full rupture of the cysts did not occur since all were still visible on the follow-up MRI. The dog also never received nonsteroidal anti-inflammatory drugs, only prednisone. In humans, intra-articular steroid injection seems to have short-term benefits such as relief of pain and clinical signs; however, long-term follow-up has shown failure rates around 60% at 6 months [13–15]. In humans, there is a report of a successful management of an atlantoaxial cyst with a neck brace and a short course of non-steroidal anti-inflammatory drugs and prednisone [11]. Unfortunately, it is not possible to know what effects, if any, the oral administration of prednisone would have on canine extradural intraspinal cysts since, to our knowledge, there are no reports of conservative management in dogs with extradural intraspinal cysts.

Interestingly, at the time of the follow-up MRI (3.5 years after initial scan), there was no pain elicited on palpation of the lumbar/lumbosacral region of the vertebral column. It is possible that the subsidence of pain might have been related to the regression of the cysts, as the dog was stable without the use of anti-inflammatories the past 10 months.

As for the extradural intraspinal cyst at S1–2, it is considered a rare presentation. It is much more common to find these cysts in areas of recognized higher mobility [1, 16]. Lumbarisation of S1 could have contributed to the formation of the cysts in this case, and transitional vertebrae have been said to affect motion in the affected and adjacent segment in humans [17]. German shepherds have been shown to have a significantly high prevalence of transitional vertebrae [18]. There have been reports where lumbosacral cysts have been identified in dogs with transitional vertebrae, and though there has been no specific link established, this may be a risk factor [1, 5, 19]. Interestingly, in the present case, the cyst at S1–2 was the only one that did not show any regression.

Although there was no histopathology performed of the cyst in this case, it was most likely a synovial cyst, since this is the most common type of extradural intraspinal cyst reported in dogs (Table 1).

**Table 1:** Dogs reported with extradural intraspinal cysts in the reviewed literature

Type of cyst	Location	Breed	Gender	Age (years)	Other imaging findings at cyst location	Clinical signs	References
Extradural instraspinal cyst	C2-C3	CKCS	M	2	No	Cervical pain	Harris et al., 2011 [20]
Ganglion^a^	L6-L7, L7-S1	GSD	F	6	Lumbarization of S1, Cd1 fused to sacrum	Lumbosacral pain	Webb et al., 2001 [4]
Synovial^a^	AA joint	Chihuahua	M	5	No	Cervical pain	Forterre et al., 2012 [8]
Synovial^a^	C3–C4, C4–C5, C5–C6	Mastiff	M	1.25	Degenerative changes and periarticular bone formation at articular process joints	Ataxia, tetraparesis, cervical pain	Levitski et al., 1999 [7]
Synovial^a^	C3–C4, C4–C5, C5–C6	Mastiff	M	1.5	Degenerative changes of articular process joints	Tetraparesis, ataxia, cervical pain	Levitski et al., 1999 [7]
Synovial^a^	C4-C5, C5-C6, C6-C7	Mastiff	F	2	Degenerative changes and remodelling of articular processes	Ataxia, tetraparesis, cervical pain	Dickinson et al., 2001 [2]
Synovial^a^	C4-C5, C5-C6, C6-C7	Mastiff	M	1.5	Degenerative changes and remodelling of articular processes	Ataxia, tetraparesis, cervical pain	Dickinson et al., 2001 [2]
Synovial^a^	C5-C6	Great Dane	M	1.25	Degenerative changes of articular process joints	Ataxia, tetraparesis, cervical pain	Levitski et al., 1999 [7]
Synovial^a^	C5-C6	Mastiff	M	1.25	Degenerative changes of articular process joints	Ataxia, tetraparesis, cervical pain	Levitski et al., 1999 [7]
Synovial^a^	C5-C6, C6-C7	Great Dane	F	2.2	Degenerative changes and remodelling of articular processes	Ataxia, tetraparesis, cervical pain	Dickinson et al., 2001 [2]
Synovial^a^	C5-C6, C6-C7	Mastiff	M	3	Degenerative changes and remodelling of articular processes	Ataxia, tetraparesis, cervical pain	Dickinson et al., 2001 [2]
Synovial^a^	C5-C6, C6-C8	Great Dane	F	1.6	Degenerative changes and remodelling of articular processes	Ataxia, tetraparesis, cervical pain	Dickinson et al., 2001 [2]
Synovial^a^	C6-C7	Great Dane	M	1	Degenerative changes and remodelling of articular processes	Ataxia, tetraparesis, cervical pain	Dickinson et al., 2001 [2]
Synovial^a^	L1-L2	GSD	M	9	Widespread degenerative articular process joint changes	Ataxia, paraparesis	Dickinson et al., 2001 [2]
Synovial^a^	L6-L7	Boxer	F	2	Sacralization of L7	Lumbosacral pain	Sale and Smith, 2007 [19]
Synovial^a^	L6-L7	GSD	F	6	Lumbarization of S1	Lumbar and lumbosacral pain	Sale and Smith, 2007 [19]
Synovial^a^	L7-S1	GSD	M	8	Ventral spondylosis, endplate sclerosis, disc protrusion	Lumbosacral pain	Schmökel and Rapp, 2016 [6]
Synovial^a^	L7-S1	GSD	F	6	Ventral spondylosis, endplate sclerosis, disc protrusion	Lumbosacral pain	Schmökel and Rapp, 2016 [6]
Synovial^a^	L7-S1	GSD	F	8	Ventral spondylosis, endplate sclerosis, and periarticular bone formation with degenerative changes of the articular processes	Lumbosacral pain	Forterre et al., 2006 [5]
Synovial^a^	L7-S1	GSD	M	9	Ventral spondylosis, endplate sclerosis, disc protrusion	Lumbosacral pain	Forterre et al., 2006 [5]
Synovial^a^	L7-S1	GSD	F	5	Ventral spondylosis, endplate sclerosis	Lumbosacral pain	Schmökel and Rapp, 2016 [6]
Synovial^a^	T13-L1	Border Collie	F	9	Bridging spondylosis of the thoracolumbar spine and degenerative changes at the articular process joints	Non-ambulatory paraparesis	Sale and Smith, 2007 [19]
Synovial^a^	T13-L1	GSD	F	8	Widespread degenerative articular process joint changes	Ataxia, paraparesis	Dickinson et al., 2001 [2]
Synovial^a^	T13-L1	GSP	F	10	Ventral spondylosis	Ataxia, paraparesis	Dickinson et al., 2001 [2]
Synovial^a^	T13-L1	Siberian Husky	M	8	No	Ataxia, paraparesis	Perez et al., 2000 [21]

^a^Confirmed via histology. *CKCS* Cavalier King Charles Spaniel, *GSD* German Shepherd Dog, *GSP* German Shorthaired Pointer

It is worth noting that by measuring each cyst at its largest diameter, there may have been an under or overestimation of the cyst reduction; however, this seemed the best alternative to quantifying these cysts since the available MR images do not allow for three-dimensional reconstruction.

The thoracolumbar region of the dog was not investigated with an MRI. The combined cervical and lumbar studies did not include T6-L1. It is thus possible that there were other extradural intraspinal cysts in that area, although there were no specific signs to suggest that, as no pain was detected on palpation on either neurological exam (first presentation or follow-up).

In summary, we report a case with multiple extradural intraspinal cysts that underwent spontaneous regression of most cysts during a 3.5-year follow-up period. Whether this is a single occurrence, or is part of the natural history of these cysts in the lumbosacral region of dogs, remains to be established.

## Data Availability

Data will be made available upon request to the corresponding author (RC_D_C).

## Abbreviations

CSM: Cervical spondylomyelopathy
MR: Magnetic resonance
MRI: Magnetic resonance imaging
T1W: T1-weighted
T2W: T2-weighted
